# Migration of stem-like CD8 T cells between tissue microenvironments underpins successful anti-tumour immune responses

**DOI:** 10.1093/discim/kyad004

**Published:** 2023-02-18

**Authors:** Bethany C Kennedy, Isaac Dean, David R Withers

**Affiliations:** Institute of Immunology and Immunotherapy, College of Medical and Dental Sciences, University of Birmingham, Birmingham, UK; Institute of Immunology and Immunotherapy, College of Medical and Dental Sciences, University of Birmingham, Birmingham, UK; Institute of Immunology and Immunotherapy, College of Medical and Dental Sciences, University of Birmingham, Birmingham, UK

**Keywords:** cancer, CD8 T cells, stem-like, immune checkpoint blockade, trafficking

## Abstract

The clinical success of immune checkpoint blockade in some patients has transformed treatment approaches in cancer and offers the hope of durable curative responses. Building from studies of chronic infection, the composition of tumour infiltrating lymphocytes and in particular, the spectrum of exhausted CD8 T cells has now been characterized in detail, profiling the phenotype, function, transcriptional regulation and even the epigenetic changes. However, what remains less clear is how intratumoural immune cells interface with populations in the periphery, both in terms of sustaining the response in cancer, but also in establishing systemic memory responses that can provide long-term protection. Here we will succinctly review the current understanding of the anti-tumour response, consider the tissue microenvironments that support key cellular subsets and the extent to which cellular migration between these sites impacts the response.

## Graphical Abstract

**Graphical Abstract:**
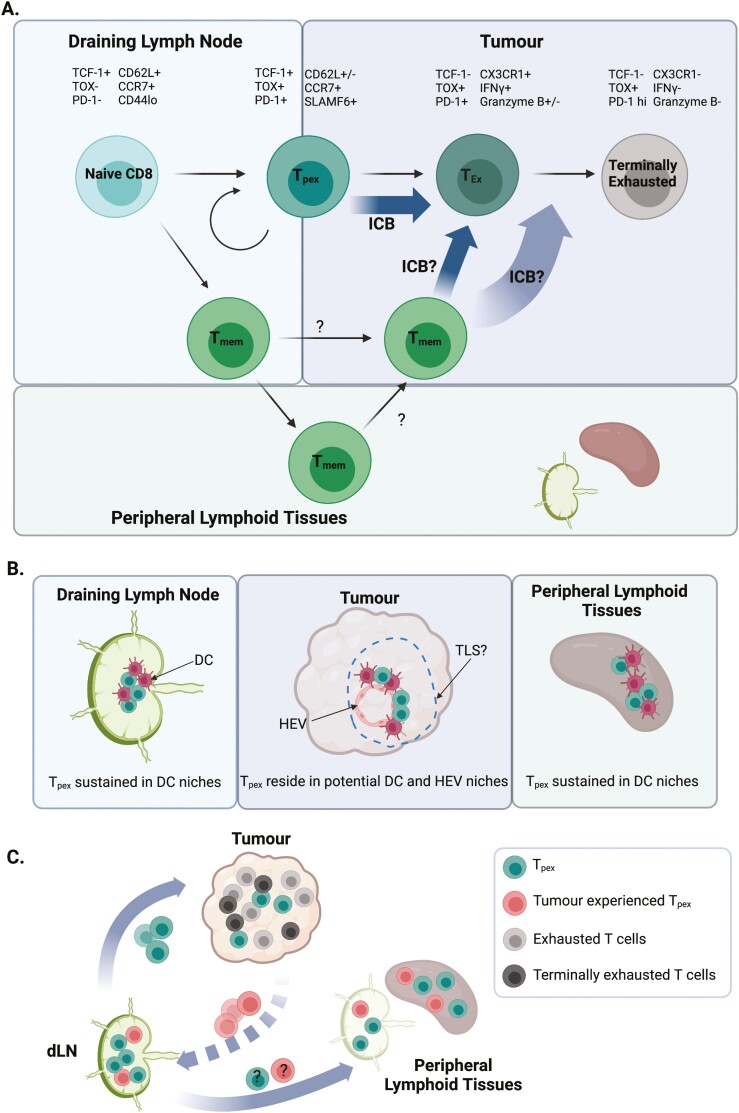

## Introduction

T cell infiltration within tumours has long been linked with improved prognosis [1]. Specifically, an increased cytotoxic CD8+ lymphocyte population is linked with better clinical outcomes across a broad range of cancer types [2–5]. The advent of immune checkpoint blockade (ICB) has been transformative in the treatment of certain cancers [6, 7]. While there remains both the need and scope to significantly refine these treatments, it has revolutionized the therapeutic paradigm for cancer patients and brought the immune response to the fore. Given that current ICB strategies work through enhancing pre-existing anti-tumour immune responses, it is unsurprising that one predictive biomarker of a response to ICB is a higher number of TILs (tumour infiltrating lymphocytes) [5, 8, 9]. While the majority of research on the anti-tumour response has focused on TILs, there is clear evidence that successful reinvigoration requires a systemic response [10, 11]. Current understanding of exactly how intratumoural T cells interface with populations in the periphery to sustain the response, but also for the development of long-term systemic protection, remains limited. Here we will succinctly review the current state of play regarding anti-tumour T cells and highlight what we understand of cellular migration between key microenvironments orchestrating this response.

### A spectrum of exhausted T cell states becomes established in tumours

Initial priming of naïve T cells requires the uptake and carriage of antigens by dendritic cells (DCs) to draining lymph nodes (dLNs). It is here that rare naïve T cells with the cognate T cell receptors are concentrated and exposed to antigen [12]; subsequent expansion of an antigen-specific T cell population underlies a successful adaptive immune response. In the context of cancer, naïve T cells must first encounter tumour antigens in the same way [13], with the cDC1 subset particularly efficient at cross-presenting antigen on MHCI to initiate CD8 T cell responses [14, 15]. As well as adequate exposure to tumour antigen, the quantity of antigen matters, with immunogenic tumours with a higher mutational burden being more responsive to immunotherapy [16]. Activated CD8 T cells then traffic to the tumour to mediate killing of the cancer cells. Unlike acute responses to infection or vaccination, where the immunological insult is cleared over a matter of days, growing tumours have by definition escaped immune control and drive sustained exposure to tumour antigens and a chronic response. This raises the possibility of further cross-priming within the tumour microenvironment (TME) should naïve T cells accumulate here, and although there is evidence to support this mechanism, its importance remains unclear [17]. The initial activation of the CD8 T cell response requires the interplay of multiple innate immune populations as well as CD4 T cell help. This has been excellently reviewed in a recent publication [18] and is beyond the focus of this review.

**Figure 1:**
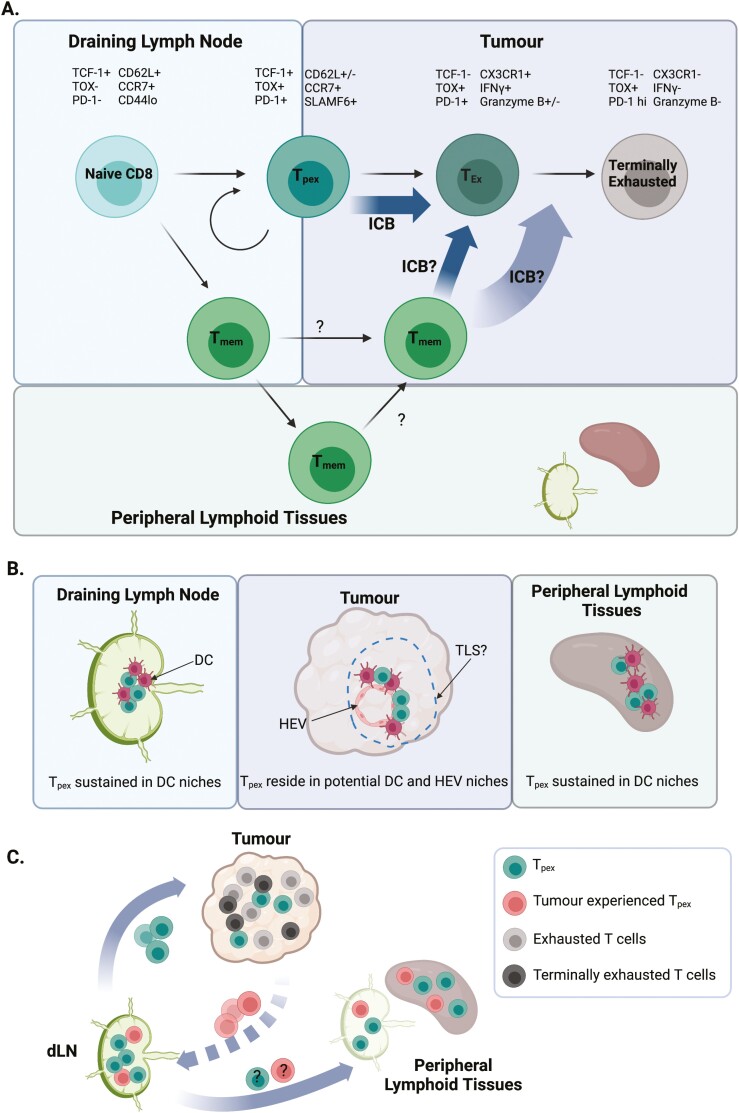
Summary of the current understanding of stem-like CD8 T cell biology in cancer. (A) *Parallel development of anti-tumour exhausted and memory CD8 T cells, their location within different tissue compartments and their roles in the response to ICB.* Naïve T cells encounter tumour antigen presented by DCs within the draining lymph node, initiating the anti-tumour immune response. Stem-like CD8 T cells (also called T_pex_), have been identified both within supportive niches enriched for DCs in the tumour, but also the tumour-draining lymph node; this population gives rise to the exhausted spectrum seen within tumours, allowing for prolonged responses to immunotherapy. Crucially, ICB drives the expansion of the stem-like compartment to replenish the exhausted cells. Memory cells, likely generated alongside the effector T cells within the dLN, seed the periphery but with the failure to clear cancer cells, become involved in the response, a process further enhanced by ICB. (B) *Tissue niches currently identified in the maintenance of stem-like CD8 T cells.* A DC niche comprised of cDC1 has been identified within secondary lymphoid tissue and within tumours. In tumours, this may reflect the presence of lymphoid aggregates within the spectrum of tertiary lymphoid structures. (C) *Stem-like CD8 T cell trafficking between tumour and lymphoid tissues.* Created using Biorender.com.

The key feature of the anti-tumour response is the accumulation of exhausted T cells within the tumour [19], a cellular state best characterized in the response to chronic viral infection [20]. The canonical features of T cell exhaustion include increased expression of co-inhibitory receptors (e.g. PD-1, TIM-3, LAG-3, and TIGIT), decreased effector functions (e.g. reduced IFNγ and TNFα production) and impaired proliferative capacity [21]. Importantly, exhaustion is a spectrum of states (illustrated in Fig. 1A), orchestrated by the transcription factor thymocyte selection-associated HMG BOX (TOX), and epigenetic commitment, defined by irreversible changes to the chromatin landscape [22, 23]. Considering the linear differentiation of T cells along this fate and the evidence that memory cells do not form during chronic infection [24], this raised the basic question of how such exhausted populations are sustained long term. Building on the evidence that antigen-specific CD8 T cells formed in chronic infection expand upon transfer into naïve hosts [25], the conundrum was resolved with the identification of a subset of exhausted cells that expressed the memory-associated transcription factor T cell factor 1 (TCF-1, encoded by *Tcf7*) [26, 27].

This stem-like CD8 population, also referred to as the ‘precursors of exhausted’ T cells (T_pex_), shared many features of memory cells (CD62L, CCR7, and CD127), lacked expression of effector genes such as Granzyme B, but also demonstrated hallmarks of exhaustion including PD-1 and LAG-3 expression [26]. Crucially, the TCF-1+PD-1+ subset of CD8 T cells in lymphocytic choriomeningitis virus (LCMV)-infected mice was responsible for sustaining the response and the proliferative burst resulting from targeting of the PD-1:PD-L1 pathway [26, 28], thus driving replenishment of the exhausted CD8 T cell compartment. Evidence for a similar cellular compartment underpinning the response to ICB in cancer patients emerged through transcriptomic profiling of clinical melanoma samples, where *Tcf7* expression within infiltrates was indicative of a positive outcome in those treated with ICB [29]. Critical further studies identified a role for TCF-1+PD-1+ CD8 T cells in driving the proliferative response in tumours following ICB therapy and the essential role of TCF-1 in this process [30].

Thus, maintenance of the exhausted T cell compartment within tumours requires a subset of exhausted cells with memory-like characteristics and crucially, these cells also proliferate in response to ICB to drive the re-expansion of exhausted, but not terminally exhausted effector cells that enhance tumour control.

### Stem-like CD8 T cell location and behaviour

While there is now consensus that the stem-like CD8 T cell subset sustains intratumoural exhausted T cells, where these cells reside and the exact microenvironments in which ICB impacts these cells, remains under debate. As we try to enhance the effect of ICB through combinatorial therapies, the basic biology of this T cell subset becomes pertinent in considering optimal strategies, as well as in the potential stratification of patients for such treatments. Multiple studies have clearly indicated the presence of stem-like cells within tumours themselves [28, 30]. A specific antigen-presenting cell (APC) niche within tumours that supports the stem-like compartment has been proposed, with TCF-1+ CD8+ T cells clustering in areas enriched for DCs and high for MHCII expression within the tumour; these areas were further associated with blood and lymphatic vascularization [31]. The presence of stem-like populations and these APC niches was associated with the prevalence of exhausted CD8 T cells as well as enhanced control of tumour growth [31]. Recent studies have highlighted the role of cDC1 in forming a niche that supports the maintenance of stem-like CD8 T cells in chronically infected lymphoid tissue, indicating that these cellular associations are a key feature for sustaining exhausted T cell responses [32]. *In vivo* imaging of the tumour to assess the impact of targeting PD-1, revealed initial DC:CD8 T cell crosstalk as a crucial initial step, suggesting that interactions within APC niches may both sustain the stem-like compartment but also drive the response to ICB [33]. In addition, Hua *et al.* recently identified a further potential niche for stem-like CD8 T cells in the proximity of tumoural high endothelial venules (HEVs), formed in response to antiangiogenic immunotherapies [34]. Exactly where and how stem-like CD8 T cell populations are sustained remains a key area of research given their role in driving the response to immunotherapy. The impact of therapeutic interventions on this cellular compartment and the effect of remodelling the tumour environment on the mechanisms that support these cells need to be resolved. Tertiary lymphoid structures (TLS), known to correlate with enhanced anti-tumour immunity, facilitate the accumulation of DCs and are associated with HEVs [35, 36], suggesting a potential link with sustaining stem-like CD8 T cells. However, tumor lysis syndrome in cancer is a spectrum of immune cell aggregates rather than a single defined state [35, 36]. Whether this reflects a linear evolution as these structures ‘mature’ or distinct cellular accumulations that support different aspects of the immune response requires further investigation. The described niches for stem-like CD8 T cells are illustrated in Fig. 1B.

Despite the presence of stem-like CD8 T cells within tumours and their assumed ability to then sustain the response from this environment, there is evidence supporting the reinvigoration of the tumour response driven by differentiation within the dLN. Dammeijier *et al.* explored the impact of targeting the tumour dLN specifically in a murine peritoneal tumour model [37]. Limited dosing of anti-PD-L1 antibodies that specifically targeted the dLN led to increased numbers of T_pex_ within this site, and despite therapy not being directed at the tumour itself, tumour regression was still observed. The authors proposed that T_pex_ generated through blocking the PD-1/PD-L1 axis were then able to seed the tumour and give rise to the anti-tumour effects observed, providing cellular links between the intratumoural and systemic compartments in the ICB response [37]. Furthermore, Dammeijier and colleagues found that it was PD-1/PD-L1 within dLNs of melanoma patients that predicted a response to ICB rather than PD-1/PD-L1 within the tumour itself [37]. Recent elegant studies utilising a mouse model of human lung adenocarcinoma indicated that a reservoir of stem-like CD8 T cells was retained in the dLN and tumour regression required cellular egress from this site [38]. These data indicate that despite the self-renewing capacity of the stem-like compartment, the intratumoural population of these cells is maintained by cellular traffic from draining lymphoid tissue. To directly assess the movement of cells into and out of tumours, we pioneered the use of the photoconvertable transgenic Kaede mouse [39], to temporally label the entire tumour immune compartment [40]. Crucially, these data revealed that while CD8 T cells retained in the tumour over a few days develop an exhausted phenotype, specific TCF-1+ populations including the stem-like subset, egressed to the dLN via the afferent lymphatics [40]. Furthermore, in studies just published, a two-step maturation of the anti-tumour response is proposed, with stem-like cells acquiring an effector state only after trafficking to the tumour, rather than full activation within the dLN [41]. Again, considering our own data generated within the Kaede mice, this two-step maturation within the tumour is consistent with the prevalence of TCF-1+ CD8 T cells amongst those newly arriving into the tumour and the subsequent emergence of an exhausted effector state [40]. Through tracking the fate of intratumoural CD8 T cells and also screening the cells that egress, our data indicate that other than the stem-like subset, the exhausted CD8 T cells remain anchored within the TME. While further studies are needed to confirm these observations, at present we see very little evidence of exhausted effector cells trafficking between the tumour and lymphoid tissue compartments.

Combined, these data clearly indicate that in murine pre-clinical models, the stem-like niche within tumours is dynamic with the continuous traffic of T_pex_ between sites. Given the identification of the supportive niche for the stem-like CD8 T cells within lymphoid tissue [32], this cellular traffic may be important for specific signals that support the long-term maintenance of this compartment. In chronic LCMV infection, parabiotic mice have indicated that the stem-like compartment remains resident within lymphoid tissue [42]. However, the systemic nature of LCMV infection makes the comparison with the cellular dynamics of a local tumour response challenging. Whether all stem-like CD8 T cells are equal in their migratory capacity remains to be determined and may explain the apparent differences in the tissue residency of these cells when LCMV infection and tumour models are compared. Building on the initial characterization of this key CD8 subset, functional heterogeneity, particularly regarding trafficking should be investigated. Noteworthy recent studies in LCMV-infected mice have also indicated that discrete regions of the lymph node (LN) favour the differentiation of short-lived effectors versus stem-like CD8 T cells [43]. Better resolution of how and where stem-like populations are initially formed within the tumour-draining LN is needed. Finally, it remains to be determined whether stem-like CD8 T cells are altered by time within the TME. Should this be the case, then further heterogeneity within the dLN stem-like progenitor pool may also develop during the anti-tumour response as migratory tumour-experienced stem-like CD8 T cells arrive in this tissue. Our current understanding of stem-like CD8 T cell trafficking and heterogeneity is summarized in Fig. 1C.

### Memory responses to tumours

The ultimate goal of ICB therapy is to achieve durable tumour regression. As in vaccination, long-term protection against cancers should require memory cells. Two questions feel most pertinent with regard to memory cells and the anti-tumour response. Firstly, are exhausted CD8 T cells able to contribute to immunological memory? Secondly, despite the focus on exhausted CD8 T cell populations, do *bona fide* memory T cells contribute to the anti-tumour response and if so, how do these roles overlap and complement the exhausted T cell response?

The long-term fate of exhausted CD8 T cells has been recently investigated in elegant studies by Abdel-Hakeem *et al.*, who exploited LCMV models to investigate what happened to exhausted CD8 T cells when chronic antigen stimulation was removed. Through comparing exhausted CD8s with memory CD8s recognizing the same LCMV epitope, but generated through acute LCMV infection, the authors discovered that even after long-term isolation from cognate antigen, the exhausted cells remained functionally impaired in their ability to mount recall responses [23]. Underpinning this, was the failure to reconfigure the epigenetic landscape from that established through exhaustion, a mechanism the authors termed ‘epigenetic-scarring’. The wider implication of this is that the exhausted T cell compartment within tumours may be poorly equipped to provide future protection against disease recurrence at the same site post-treatment or at a distant metastatic site. However, the TCF-1+ subset of exhausted CD8 T cells appeared to be the least ‘scarred’ in terms of persisting post-transfer, raising the potential for this subset to provide memory-like functions after tumour resolution [23]. Returning to our observation that the stem-like subset of CD8 T cells egressed to the dLN [40], it is tempting to speculate that tumour-experienced stem-like cells may become circulatory and thus potentially contribute to systemic surveillance. The initial characterization of T_pex_ identified their expression of the pathways required to circulate through secondary lymphoid tissue [25]. Recent studies highlighted the presence of a CD62L+ subset of stem-like CD8s within the tumour that showed the greatest proliferative capacity in response to PD-1 blockade [44]. Thus, functional heterogeneity appears evident within the different stem-like fractions and this may extend to distinct migratory capacities.

There is increasing evidence that alongside the exhausted CD8 T cell compartment in tumours, both memory CD8 and CD4 T cells contribute to the enhanced anti-tumour response induced by ICB [10, 45, 46]. Since the response fails to clear the tumour, rather than only seeding the periphery to protect against subsequent re-encounter, memory cells may become recruited into the response. Given current evidence that exhausted T cells are impaired in their ability to form memory cells [23], it seems most likely that memory CD8 cells are formed in parallel (Fig. 1A). Investigations of the systemic response to ICB further indicate the importance of memory cells within peripheral lymphoid tissues [10]. Single-cell RNA sequencing across four different types of human cancer, linked an expanded peripheral T cell pool to T cell infiltrate in tumours and the subsequent response to immunotherapy [11]. These studies hinted that non-exhausted T cell clones in the blood may provide a key to understanding the varying patient responses to ICB and support patient stratification.

### Concluding comments

TILs are essential in successful anti-cancer immune responses. ICB enhances the anti-tumour T cell response through driving the proliferation and differentiation of stem-like CD8 T cells (also called T_pex_) to restore the exhausted T cell compartment from one dominated by terminally exhausted cells. Memory cells formed in parallel with the exhausted T cell compartment further augment this response. It seems that the movement of both early exhausted and memory T cell populations between the tumour, dLN and systemic lymphoid tissue ensures that robust anti-tumour responses are maintained. A more precise understanding of exactly which populations migrate and what this means for sustaining the response within the tumour is key. Clinically, a better understanding of the dynamics of the anti-tumour T cell response could assist in formulating more informed therapeutic combinations or improving prognostic markers.

## Data Availability

This is a review article and no new data is presented.

## Abbreviations

APC: antigen-presenting cell
DC: dendritic cell
dLN: draining lymph node
ICB: immune checkpoint blockade
LCMV: lymphocytic choriomeningitis virus
TCF-1: T cell factor 1
TILs: tumour infiltrating lymphocytes
TME: tumour microenvironment
TOX: thymocyte selection-associated HMG BOX
T_pex_: T precursors of exhausted

## Animal research statement

This is a review article and no in vivo experimentation was performed.
